# Conditional survival after neoadjuvant chemoradiotherapy and surgery for oesophageal cancer

**DOI:** 10.1002/bjs.11476

**Published:** 2020-02-03

**Authors:** E. R. C. Hagens, M. L. Feenstra, W. J. Eshuis, M. C. C. M. Hulshof, H. W. M. van Laarhoven, M. I. van Berge Henegouwen, S. S. Gisbertz

**Affiliations:** ^1^ Department of Surgery Amsterdam University Medical Centres, Location AMC, University of Amsterdam, Cancer Centre Amsterdam Amsterdam the Netherlands; ^2^ Department of Radiotherapy Amsterdam University Medical Centres, Location AMC, University of Amsterdam, Cancer Centre Amsterdam Amsterdam the Netherlands; ^3^ Department of Medical Oncology Amsterdam University Medical Centres, Location AMC, University of Amsterdam, Cancer Centre Amsterdam Amsterdam the Netherlands

## Abstract

**Background:**

Conditional survival accounts for the time already survived after surgery and may be of additional informative value. The aim was to assess conditional survival in patients with oesophageal cancer and to create a nomogram predicting the conditional probability of survival after oesophagectomy.

**Methods:**

This retrospective study included consecutive patients with oesophageal cancer who received neoadjuvant chemoradiation followed by oesophagectomy between January 2004 and 2019. Conditional survival was defined as the probability of surviving *y* years after already surviving for *x* years. The formula used for conditional survival (CS) was: CS_(*x*|*y*)_ = S_(*x* + *y*)_/S_(*x*)_, where S_(*x*)_ represents overall survival at *x* years. Cox proportional hazards models were used to evaluate predictors of overall survival. A nomogram was constructed to predict 5‐year survival directly after surgery and given survival for 1, 2, 3 and 4 years after surgery.

**Results:**

Some 660 patients were included. Median overall survival was 44·4 (95 per cent c.i. 37·0 to 51·8) months. The probability of achieving 5‐year overall survival after resection increased from 45 per cent directly after surgery to 54, 65, 79 and 88 per cent given 1, 2, 3 and 4 years already survived respectively. Cardiac co‐morbidity, cN category, ypT category, ypN category, chyle leakage and pulmonary complications were independent predictors of survival. The nomogram predicted 5‐year survival using these predictors and number of years already survived.

**Conclusion:**

The probability of achieving 5‐year overall survival after oesophagectomy for cancer increases with each additional year survived. The proposed nomogram predicts survival in patients after oesophagectomy, taking the years already survived into account.

## Graphical abstract

Conditional survival accounts for the time already survived after surgery and may be of additional informative value. A nomogram was constructed that predicts 5‐year survival of patients with oesophageal cancer after neoadjuvant treatment followed by surgery, based on cardiac co‐morbidity, cN category, ypTN stage, chyle leakage and pulmonary complications, taking years already survived into account.

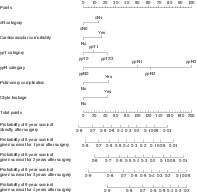

Informative for patient and doctor

## Abstract (es)

**Antecedentes:**

La supervivencia condicional hace referencia al tiempo ya sobrevivido tras la cirugía y esta información puede tener un valor adicional. El objetivo fue evaluar la supervivencia condicional en pacientes con cáncer de esófago y crear un nomograma para predecir la probabilidad condicional de supervivencia tras una esofaguectomía.

**Métodos:**

Estudio retrospectivo incluyó pacientes consecutivos con cáncer de esófago que fueron tratados con quimiorradioterapia neoadyuvante seguida de cirugía, entre enero de 2004 a 2019, en el centro médico de la Universidad de Amsterdam (AMC) de los Países Bajos. La supervivencia condicional se definió como la probabilidad de sobrevivir y años tras haber ya sobrevivido ya durante x años. La formula utilizada fue: CS_(x|y)_=S_(x+y)_/S_(x)_, con S_(x)_ representando la supervivencia global a x años. Se utilizaron modelos de riesgo proporcional de Cox para evaluar los predictores de supervivencia global. Se construyó un nomograma para predecir la supervivencia a los 5 años directamente tras la cirugía y dar la supervivencias a 1‐, 2‐, 3‐ y 4 años después de la cirugía.

**Resultados:**

Se incluyeron 660 pacientes. La mediana de la supervivencia global fue de 44,4 meses (i.c. del 95% 37,0‐51,8). La probabilidad de conseguir una supervivencia global a los 5 años tras la resección aumentó del 45% directamente después de la cirugía al 54%, 65%, 79% y 88% por cada año adicional sobrevivido**.** La comorbilidad cardiaca, estadio cN, estadio ypT, estadio ypN, quilotórax y complicaciones pulmonares fueron predictores independientes de supervivencia. El nomograma predijo la supervivencia a 5 años utilizando estos predictores y número de años ya sobrevividos.

**Conclusión:**

La probabilidad de alcanzar una supervivencia global a los 5 años tras una esofaguectomía por cáncer aumenta por cada año adicional sobrevivido. El nomograma propuesto predice la supervivencia en pacientes después de una esofaguectomía, teniendo en cuenta los años ya sobrevividos.

## Introduction

Oesophageal cancer is an aggressive disease with an increasing incidence. It is the sixth leading cause of cancer‐related death for men and women combined worldwide^1^, ^2^, ^3^. Mortality rates, however, have decreased; 5‐year overall survival was 15·3 per cent in 2000 and is 30–57 per cent at present^4^, ^5^. One factor contributing to the improvement in survival is the introduction of neoadjuvant therapy^6^, ^7^.

Most survival rates reported in the literature are static, being calculated from the day of diagnosis or surgery^8^, ^9^, ^10^, ^11^. However, the risk of death changes with time after surgery^12^. Conditional survival represents the probability of surviving a certain number of years after diagnosis or treatment based on the time the patient has already survived. Conditional survival might therefore be more meaningful for patients than conventional survival analysis as it provides a more individualized prognosis as time progresses. Physicians may adapt follow‐up visits according to the conditional survival pattern.

Apart from the time after surgery, other factors such as co‐morbidities and postoperative complications influence the chance of survival. A nomogram is based on the most important factors predicting survival. Survival nomograms for patients with oesophageal cancer have recently been developed, both for curative and palliative settings^13^, ^14^, ^15^, ^16^, ^17^, ^18^. However, current nomograms do not take into account the years that a patient has already survived.

A previous study^12^ assessed conditional survival among patients undergoing surgery for oesophageal cancer. However, it analysed a cohort of patients from 1988 to 2011 who were not treated with neoadjuvant therapy. The aim of the present study was to define conditional survival among patients undergoing surgery for oesophageal carcinoma after neoadjuvant treatment, and to design a nomogram for predicting the conditional probability of survival after oesophagectomy for cancer.

## Methods

This retrospective study was conducted at the Amsterdam University Medical Centres, location AMC, the Netherlands. Consecutive patients with oesophageal cancer who underwent oesophagectomy between January 2004 and January 2019 and who met the inclusion criteria were included. Ethical approval is not mandatory for retrospective studies in the Netherlands. All patients who were still alive at the end of the inclusion period were asked to provide consent for their data to be used (anonymously) for this retrospective study. The TRIPOD guidelines were followed to ensure the correct reporting of the results^19^.

### Study population

Included were patients with resectable (cT0–4a N0–3 M0) oesophageal adenocarcinoma or squamous cell carcinoma receiving neoadjuvant chemoradiation therapy, followed by open or minimally invasive transthoracic or transhiatal oesophagectomy with a cervical or intrathoracic anastomosis. The choice of surgical procedure depended on characteristics of the tumour (location, stage, location of lymph node metastases) and patient (co‐morbidities). Neoadjuvant therapy comprised weekly carboplatin (area under curve 2) and paclitaxel (50 mg/m^2^) for 5 weeks combined with daily radiotherapy consisting of 23 fractions of 1·8 Gy (total 41·4 Gy)^20^. This treatment was administered to patients allocated to the neoadjuvant therapy group in the CROSS study between 2004 and 2008^6^. Since 2009, neoadjuvant therapy has been the standard of care, and all patients having treatment with curative intent received neoadjuvant chemoradiotherapy unless considered unfit for multimodal treatment. Patients who did not undergo neoadjuvant chemoradiotherapy, those with distant metastases, patients who underwent salvage surgery, those who died in hospital or within 90 days after resection owing to a complication, and patients who did not provide informed consent were excluded from the study.

### Defining factors influencing long‐term survival

In January 2019, a systematic search of the available literature in online databases was undertaken to identify relevant studies describing variables influencing postoperative outcomes. The following keywords were used: oesophageal cancer, oesophagectomy, risk factors, long‐term survival, mortality and recurrence. Potential factors found to influence long‐term survival were: co‐morbidities, presence and severity of postoperative complications, tumour characteristics such as TNM stage and tumour differentiation, Tumour Regression Grade according to the Mandard classification, and resection margins^21^, ^22^, ^23^, ^24^.

### Data collection

Patient characteristics and co‐morbidities (including chronic obstructive pulmonary disease, type 2 diabetes and cardiovascular co‐morbidity), tumour characteristics, treatment details, postoperative complications and oncological outcomes were collected from a prospectively maintained database. Cardiovascular co‐morbidity included cardiac failure, arrhythmia, history of myocardial infarction, hypertension, peripheral artery occlusive disease and aortic aneurysm. Postoperative complications were scored using the definitions of Low and colleagues^25^. Factors potentially influencing long‐term survival that were not included in the prospectively maintained database were retrieved from patient files. Tumour stage was assessed using the eighth edition of the AJCC staging system^26^.

### Statistical analysis

Overall survival was calculated from the day of surgery until the day of death from any cause (event), or last day of follow‐up (censored). Median overall survival was calculated using Kaplan–Meier analysis and reported with 95 per cent confidence interval.

Conditional survival is defined as the probability of surviving an additional number of *y* years given that a patient has already survived for *x* years, and can be calculated from Kaplan–Meier survival data. Conditional survival (CS) can be expressed mathematically as: CS_(*x*|*y*)_ = S_(*x* + *y*)_/S_(*x*)_, where S_(*x*)_ represents overall survival at *x* years after surgery estimated using the Kaplan–Meier method^27^. For example, conditional survival for surviving another year among patients who had already survived 4 years, CS_(1|4)_, was calculated by dividing the 5‐year Kaplan–Meier survival estimate S_(5)_ by the 4‐year survival estimate S_(4)_ 27, 28.

Variables associated with survival were identified from the literature, as described above. Available potential predictors were all entered into a multivariable Cox proportional hazards model. In the backward elimination procedure, variables with *P* > 0·050 were excluded from the model to identify the most accurate and parsimonious set of predictors, and to increase the practical applicability of the models. Hazard ratios are reported with 95 per cent confidence intervals. Underlying assumptions for the Cox proportional hazards model were evaluated and shown to be met.

For the prediction nomogram, coefficients of the predictors in the multivariable Cox proportional hazards model were re‐estimated, using a penalized LASSO (least absolute shrinkage and selector operator) Cox proportional hazards model to prevent overfitting^27^, ^29^, ^30^. Model performance was assessed by measuring discrimination (ability to discriminate between participants with or without an event) and calibration (ability to quantify the observed absolute risk). The discriminative ability of the model was examined using the C‐statistic, which was corrected for optimistic predicting by bootstrapping (B = 200), where *P* > 0·050 indicates good performance between predicted and observed risks.

Missing data were imputed according to predictive mean matching principles, using 20 imputations. All *P* values were based on a two‐sided test and *P* < 0·050 was considered statistically significant. Data were analysed using SPSS® for Windows® version 25 (IBM, Armonk, New York, USA) and R version 3.3.3 (R Foundation for Statistical Computing, Vienna, Austria).

## Results

**Figure 1:**
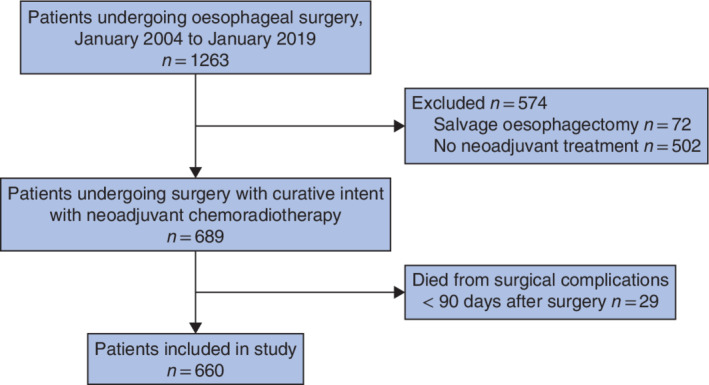
Study flow chart

In total, 1263 patients underwent surgery for oesophageal carcinoma between January 2004 and January 2019. Some 574 patients were excluded because they did not meet the inclusion criteria and 29 patients died in hospital or within 90 days after surgery from a complication, leaving 660 eligible patients for inclusion in this study (*Fig*. 1). Some 523 patients had an adenocarcinoma and 137 had a squamous cell carcinoma. By 1 March 2019, 316 patients had died, 268 from an oesophageal cancer‐related cause. Characteristics of the patients are shown in *Tables* 1 and 2.

**Table 1:** Baseline and treatment characteristics

	No. of patients (*n* = 660)
**Age ≤ 64 years**	340 (51·5)
**Men**	528 (80·0)
**BMI > 25 kg/m** ^**2**^	346 (52·4)
Missing	8 (1·2)
**Cardiovascular co‐morbidity**	248 (37·6)
**COPD**	38 (5·8)
**Type 2 diabetes mellitus**	71 (10·8)
Missing	3 (0·5)
**ASA fitness grade**	
I	230 (34·8)
II	315 (47·7)
III	114 (17·3)
Missing	1 (0·2)
**Clinical T category**	
cT1	6 (0·9)
cT2	134 (20·3)
cT3	498 (75·5)
cT4	5 (0·8)
Missing	17 (2·6)
**Clinical N category**	
cN0	201 (30·5)
cN1	347 (52·6)
cN2	99 (15·0)
cN3	9 (1·4)
Missing	4 (0·6)
**Neoadjuvant treatment completed**	650 (98·5)
**Adjuvant therapy**	37 (5·6)
**Transthoracic approach**	561 (85·0)
**Minimally invasive oesophagectomy** *	456 (69·0)
**Cervical anastomosis**	382 (57·9)
Missing	4 (0·6)

Values in parentheses are percentages.

* Two patients underwent hybrid minimally invasive surgery; other procedures were fully minimally invasive. COPD, chronic obstructive pulmonary disease.

**Table 2:** Histopathological data and complications

	No. of patients (*n* = 660)
**Histology**	
Squamous cell carcinoma	137 (20·8)
Adenocarcinoma	523 (79·2)
**R0 resection**	635 (96·2)
**Pathological T category**	
ypT0	133 (20·2)
ypT1	108 (16·4)
ypT2	103 (15·6)
ypT3	288 (43·6)
ypT4	4 (0·6)
Missing	24 (3·6)
**Pathological N category**	
ypN0	398 (60·3)
ypN1	148 (22·4)
ypN2	77 (11·7)
ypN3	37 (5·6)
**Tumour regression grade (Mandard score)**	
TRG 1	157 (23·8)
TRG 2	131 (19·8)
TRG 3	177 (26·8)
TRG 4	108 (16·4)
TRG 5	35 (5·3)
Missing	52 (7·9)
**Postoperative complications**	
Pulmonary complications	178 (27·0)
Cardiac complications	135 (20·5)
Anastomotic leakage	83 (12·6)
Chyle leak	71 (10·8)
Sepsis	21 (3·1)
**Clavien–Dindo grade**	
0	274 (41·5)
I	98 (14·8)
II	109 (16·5)
IIIa	60 (9·1)
IIIb	16 (2·4)
IVa	75 (11·4)
IVb	28 (4·2)

Values in parentheses are percentages. TRG, Tumour Regression Grade.

### Conditional overall survival

**Figure 2:**
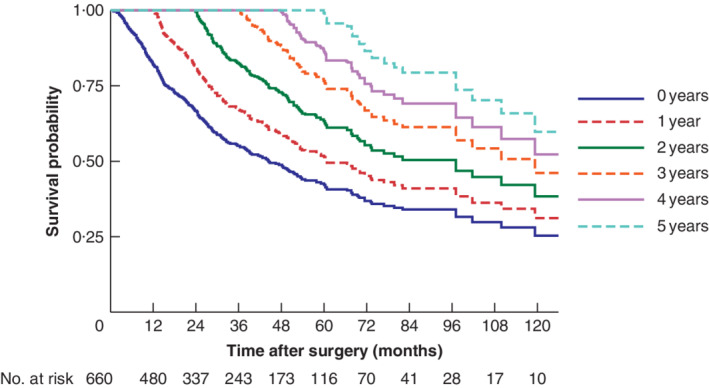
Kaplan–Meier estimates of survival after surgery (0 years) and conditional survival, according to years already survived after surgery (1–5 years)

Median overall survival was 44·4 (95 per cent c.i. 37·0 to 51·8) months, with a 5‐year survival rate of 45 per cent. Conditional overall survival probabilities are shown in *Table* 3 and survival curves in *Fig*. 2, in relation to the number of years already survived after surgery. The probability of achieving 5‐year survival after resection increased from 45 per cent directly after surgery to 54, 65, 79 and 88 per cent given 1, 2, 3 and 4 years already survived respectively. The more years patients had already survived, the better their chances of additional years of survival. This increase flattened after more years had passed. For example, the 2‐year conditional survival (probability of surviving the next 2 years) was 69 per cent directly after surgery, 74 per cent after 2 years, 78 per cent after 4 years and 92 per cent after 6 years.

**Table 3:** Conditional overall survival estimates

Total years of survival after surgery	Probability of survival (%)							
	Years already survived by patient							
	0	1	2	3	4	5	6	7
1	83							
2	69	83						
3	57	68	83					
4	51	61	74	89				
5	45	54	65	79	88			
6	40	48	58	70	78	89		
7	37	44	54	65	72	82	93	

The probability of survival after surgery is shown in relation to the number of years already survived. For example, if a patient has survived 2 years after surgery, the probability of achieving 3‐year survival after surgery is 83 per cent and of achieving 5‐year survival after surgery is 65 per cent.

### Predictors of overall survival

In univariable analysis, decreased overall survival was associated with cardiovascular co‐morbidity, type 2 diabetes, a higher cT or cN category, R1 resection, higher ypT or ypN category, a higher Mandard score, presence of chyle leakage, any pulmonary complication and an increased Clavien–Dindo grade (*Table* 4). Cardiovascular co‐morbidity, cN, ypN and ypT category, chyle leakage and pulmonary complications were significant predictors of overall survival in multivariable analysis after backward selection (*Table* 4). In separate analyses of predictive factors for overall survival of patients with adenocarcinoma and squamous cell carcinoma, ypN category remained a strong predictor for both tumour types. Cardiovascular co‐morbidity, chyle leakage and pulmonary complications remained as significant predictors of impaired survival in the final model in patients with adenocarcinoma, whereas R1 resection and higher Mandard score were significant predictors of impaired survival in patients with squamous cell carcinoma (*Table* *S1*, supporting information).

**Table 4:** Univariable and multivariable Cox proportional hazards analysis of risk factors associated with overall survival

	Univariable analysis		Multivariable analysis*		
	Hazard ratio	*P*	β	Hazard ratio	*P*
**Age > 64 years**	1·04 (0·84, 1·30)	0·794			
**Male sex**	1·11 (0·84, 1·48)	0·447			
**BMI > 25 kg/m** ^**2**^	1·00 (0·80, 1·25)	0·999			
**Cardiovascular co‐morbidity**	1·29 (1·03, 1·61)	0·026	0·311	1·37 (1·08, 1·71)	0·008
**COPD**	1·17 (0·77, 1·79)	0·460			
**Type 2 diabetes mellitus**	1·45 (1·05, 2·02)	0·026			
**ASA fitness grade**					
I	1·00 (reference)				
II	1·15 (0·90, 1·47)	0·256			
III	1·19 (0·87, 1·64)	0·280			
**Transthoracic oesophagectomy**	0·95 (0·72, 1·26)	0·740			
**Minimally invasive approach**	1·05 (0·83, 1·32)	0·678			
**cT3 (*versus* cT2)**	1·63 (1·19, 2·23)	0·002			
**cN+**	1·47 (1·14, 1·89)	0·003	0·265	1·31 (1·01, 1·69)	0·044
**Squamous cell carcinoma (*versus* adenocarcinoma)**	0·79 (0·60, 1·04)	0·087			
**R0 resection**	0·35 (0·22, 0·55)	< 0·001			
**Pathological T category** †					
ypT0	1·00 (reference)			1·00 (reference)	
ypT1	1·39 (0·92, 2·11)	0·121	0·174	1·19 (0·79, 1·80)	0·411
ypT2–T3	2·24 (1·62, 3·11)	< 0·001	0·404	1·50 (1·07, 2·11)	0·020
**Pathological N category**					
ypN0	1·00 (reference)			1·00 (reference)	
ypN1	2·52 (1·93, 3·30)	< 0·001	0·918	2·51 (1·89, 3·31)	< 0·001
ypN2	3·53 (2·59, 4·82)	< 0·001	1·141	3·14 (2·25, 4·36)	< 0·001
ypN3	6·95 (4·67, 10·34)	< 0·001	1·843	6·34 (4·16, 9·58)	< 0·001
**Tumour Regression Grade**					
TRG 1–2	1·00 (reference)				
TRG 3	1·47 (1·11, 1·97)	0·008			
TRG 4–5	2·35 (1·79, 3·10)	< 0·001			
**Postoperative complications**					
Pulmonary complication	1·33 (1·05, 1·68)	0·017	0·422	1·53 (1·20, 1·94)	< 0·001
Cardiac complication	1·05 (0·80, 1·38)	0·759			
Anastomotic leakage	0·88 (0·63, 1·24)	0·474			
Chyle leakage	1·47 (1·06, 2·05)	0·021	0·408	1·51 (1·07, 2·11)	0·018
Clavien Dindo grade ≥ IIIb	1·44 (1·11, 1·88)	0·006			

Values in parentheses are 95 per cent confidence intervals.

* Coefficients in multivariable model calculated from LASSO model.

† ypT4 patients not included in the analysis due to the small group size. COPD, chronic obstructive pulmonary disease.

### Conditional survival stratified by disease stage, cardiovascular co‐morbidity, postoperative pulmonary complications and chyle leakage

Stratification of conditional survival by stage of disease showed greater improvement in conditional survival in patients with more advanced disease (*Fig*. *S1*, supporting information). The effect of pulmonary complications on overall survival was constant over time, whereas the effects of cardiovascular co‐morbidities and chyle leakage were greater in the first years after surgery and then flattened.

### Prediction nomogram for conditional overall survival

**Figure 3:**
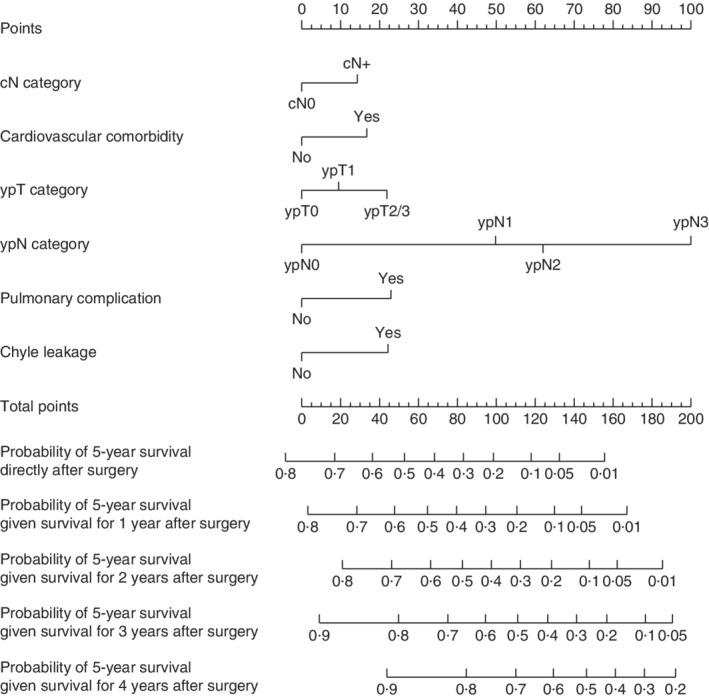
Nomogram predicting probability of achieving 5‐year survival after surgery for oesophageal cancer cN, ypT and ypN categories according to AJCC 8th edition.

Cardiovascular co‐morbidity, cN, ypN and ypT category, chyle leakage and pulmonary complications were included in the nomogram. The baseline survival function So_(*t*)_ was 0·768 for 5 years. The prediction model had an optimism‐adjusted C‐statistic of 0·70 (95 per cent c.i. 0·69 to 0·70). *Fig*. *S2* (supporting information) shows the calibration plot of the nomogram; there was high correspondence between predicted and actual survival. The nomogram, which predicts the probability of reaching 5‐year survival directly after surgery and if the patient has already survived for 1–4 years after operation, is shown in *Fig*. 3. For example, a patient with cardiovascular co‐morbidity, cN+, ypT1 and yN2 categories, without any postoperative complications, has a total nomogram score of 105 points. The probability of 5‐year survival is 18 per cent directly after surgery, which increases to 55 per cent if the patient survives the first 3 years after surgery. To facilitate user‐friendly calculations, the probability of achieving 5‐year survival, given *x* years of survival after surgery, was added to an online calculator, available at http://www.uppergi.nl/webcalculator.

## Discussion

In this study, conditional overall survival probability following neoadjuvant chemoradiotherapy and oesophagectomy was evaluated in patients with oesophageal carcinoma, and a nomogram was developed that can be used to provide patients and physicians with accurate information about prognosis. Using this chart, patients can be shown visually that, when more time has passed, the chances of surviving oesophageal cancer increases. The longer the interval that a patient has survived after surgery, the higher the chance of surviving an additional year. The C‐statistic and calibration plot indicated that the nomogram predictions are accurate; it is therefore a useful tool for predicting outcomes during follow‐up after oesophageal cancer surgery.

Conditional survival considers the number of years that a patient has already survived when estimating the survival probability. The probability of surviving 5 years after surgery changes from 45 per cent if 0 years are survived to 79 per cent if 3 years are survived. Conditional survival is therefore a valuable addition to the prediction of survival after treatment for oesophageal cancer, as shown in studies of conditional survival in other malignancies^27^, ^28^, ^31^.

Cardiovascular co‐morbidities, cN, ypT and ypN categories, chyle leakage and pulmonary complications were independent predictors of survival in the present analysis. Shapiro and colleagues^32^ developed a prediction model for survival of patients with oesophageal cancer after neoadjuvant chemoradiation, and also found cN, ypT and ypN categories to be strong predictors of survival. In contrast to the present study, conditional survival was not implemented in their final model and postoperative complications were not included in the analyses. Other studies have shown that patients who developed severe postoperative complications had impaired long‐term survival compared with those who did not have postoperative complications. In accordance with the present findings, chyle leakage and pulmonary complications have previously be identified as independent predictors for reduced long‐term survival in patients with oesophageal carcinoma^24^, ^33^, ^34^. It can be argued whether or not complications themselves lead to worse survival, or whether these patients already have more co‐morbidities that cause complications and their survival is impaired because of that. Postoperative complications are often not considered when prediction models for overall survival are developed because the aim is usually to enable preoperative prediction of survival. However, in the case of conditional survival, the survival probability is estimated after surgery and it is important to consider postoperative factors.

The final nomogram included cN, ypT and ypN categories, cardiovascular co‐morbidities, postoperative chyle leakage and pulmonary complications. The conditional survival probability can be calculated manually using the nomogram presented in *Fig*. 3 or by using the online calculator^35^. This nomogram has a C‐statistic of 0·70, which indicates good performance of this model compared with other oncological prediction models. Similar models predicting survival in patients with oesophageal cancer after neoadjuvant treatment and surgery have C‐statistic values ranging from 0·61 to 0·72^13^, ^16^, ^36^. Moreover, the calibration plot shows good correspondence between predicted and observed survival, indicating that the prediction nomogram is accurate.

This study has some limitations. As it is a retrospective analysis, some variables that might have an influence on survival were missing from the database and could not be integrated in the Cox regression models. For example, Zhang^37^ used the US Surveillance, Epidemiology, and End Results database to show that patients of African descent have higher mortality rates than Caucasians. Another important variable is tumour differentiation. In an evaluation of factors associated with early recurrence and death after oesophagectomy for cancer, Davies and co‐workers^23^ found that poor tumour differentiation was a significant independent marker of worse survival in a multivariable model. This variable was missing from most of the patient files and could not be included in the present analysis. A further limitation was that patients who were operated most recently had relatively short follow‐up. Therefore, the groups analysed for conditional survival during the later years were smaller. Finally, the results of this study are only applicable to patients treated with neoadjuvant chemoradiotherapy. Not all patients undergo neoadjuvant chemoradiotherapy as part of treatment with curative intent as treatment guidelines differ around the world.

Several prediction models including nomograms have been designed to predict survival of patients with oesophageal cancer treated with curative intent^14–16,32^. The present model is based on data from a single high‐volume centre. Individual‐surgeon numbers of resections were not available. Future studies are needed to externally validate and update this prediction model, and make it more generalizable to smaller centres. A large study could identify whether other variables such as tumour differentiation are predictors of overall survival and could add value to a prediction model for overall survival.

## Supporting information


**Appendix S1.** Supporting Information
