# Supplementary material for: Conditional survival after neoadjuvant chemoradiotherapy and surgery for oesophageal cancer
Source: Br J Surg. 2020 Feb 3;107(8):1053–61. doi: 10.1002/bjs.11476 (PMC7317937; doi:10.1002/bjs.11476)

**BJS11476**

**Conditional survival after curative treatment of oesophageal cancer**

E. R. C. Hagens, M. L. Feenstra, W. J. Eshuis, M. C. C. M. Hulshof, H. W. M. van Laarhoven, M. I. van Berge Henegouwen and S. S. Gisbertz

| **Table S1 Multivariable analysis for risk factors associated with overall survival according to the Cox Proportional Hazard model for adenocarcinoma and squamous cell carcinoma** | | | | | | | | | | | | |
| --- | --- | --- | --- | --- | --- | --- | --- | --- | --- | --- | --- | --- |
|  | **Adenocarcinoma** | | | | | | **Squamous cell carcinoma** | | | | | |
|  |  | | **95% CI** | |  |  | |  | **95% CI** | | |  |
|  | **Hazard Radio** | **Lower limit** | | **Upper limit** | **P-value** |  | | **HR** | | **Lower limit** | **Upper limit** | **P-value** |
| Cardiovascular comorbidity | 1.448 | 1.125 | | 1.863 | 0.004 |  | |  | |  |  |  |
| No R0 resection |  |  | |  |  |  | | 4.948 | | 1.496 | 16.370 | 0.009 |
| Pathological N stage |  |  | |  |  |  | |  | |  |  |  |
| ypN0 | *reference* |  | |  |  |  | | *reference* | |  |  |  |
| ypN1 | 2.854 | 2.102 | | 3.875 | <0.001 |  | | 2.436 | | 1.288 | 4.608 | 0.006 |
| ypN2 | 3.895 | 2.769 | | 5.479 | <0.001 |  | | 3.618 | | 1.375 | 9.521 | 0.009 |
| ypN3 | 7.912 | 5.159 | | 12.132 | <0.001 |  | | 2.211 | | 0.299 | 16.318 | 0.437 |
| Tumor regression grade |  |  | |  |  |  | |  | |  |  |  |
| TRG 1-2 |  |  | |  |  |  | | *reference* | |  |  |  |
| TRG 3 |  |  | |  |  |  | | 1.192 | | 0.604 | 2.351 | 0.612 |
| TRG 4-5 |  |  | |  |  |  | | 2.547 | | 1.187 | 5.467 | 0.016 |
| Postoperative complications |  |  | |  |  |  | |  | |  |  |  |
| Chyle leak | 1.733 | 1.186 | | 2.531 | <0.001 |  | |  | |  |  |  |
| Pulmonary complication | 1.553 | 1.189 | | 2.029 | <0.001 |  | |  | |  |  |  |
| *CI = confidence interval, TRG = tumor regression grade* | | | | | | | | | | | | |

**Fig. S1 Five-year conditional overall survival of patients with esophageal cancer by a) grade, b) presence of cardiovascular comorbidity, c) pulmonary complications and d) postoperative chyle leak**

**a**

**
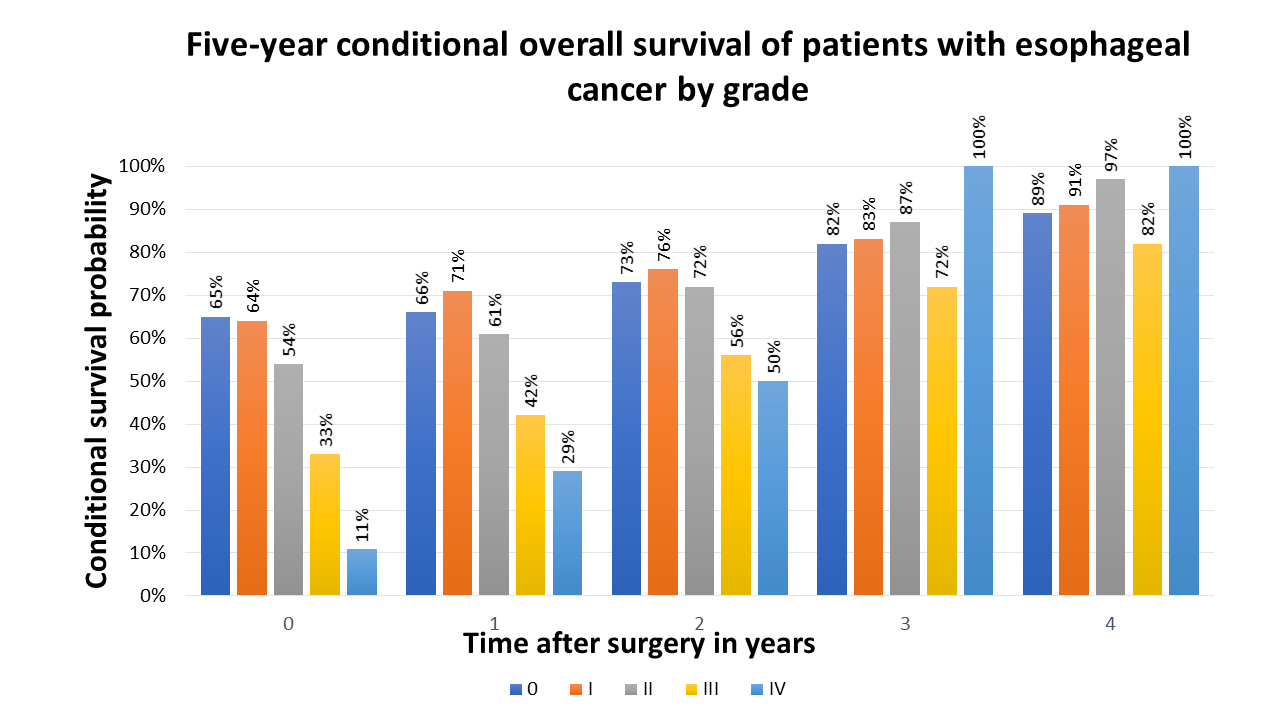
**

**b**

**
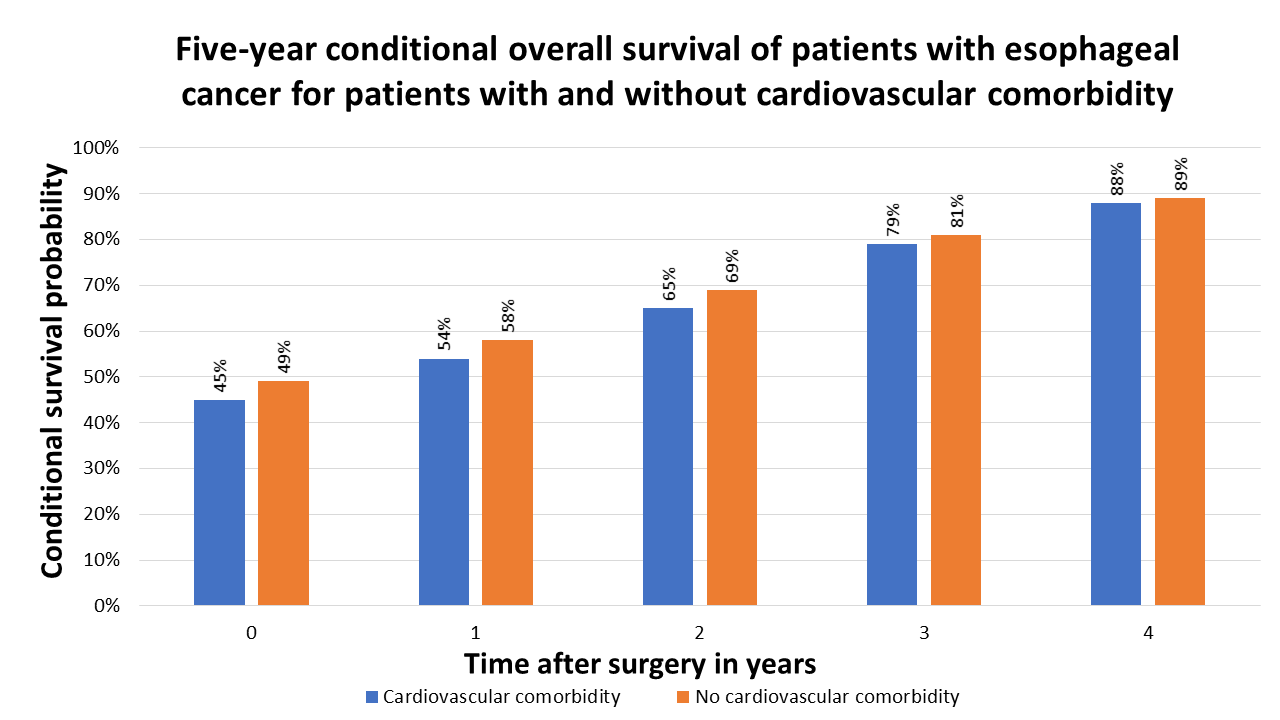
**

**c**

**
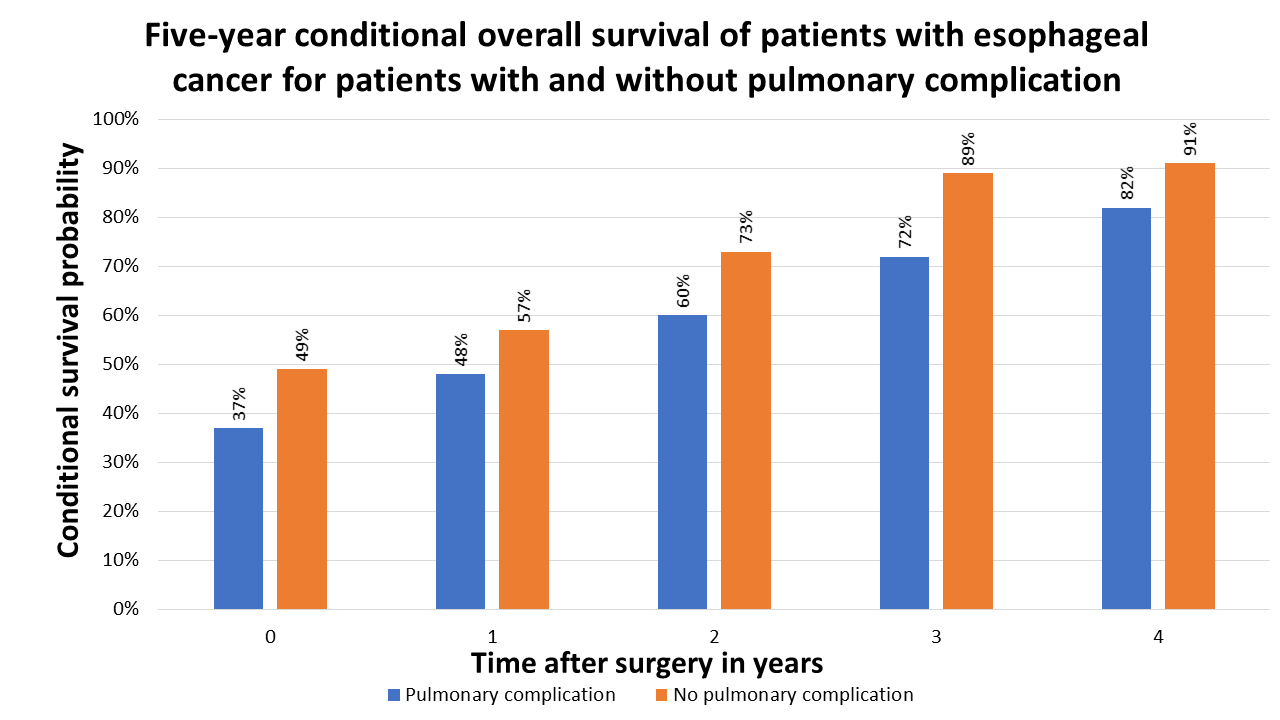
**

**d**

**
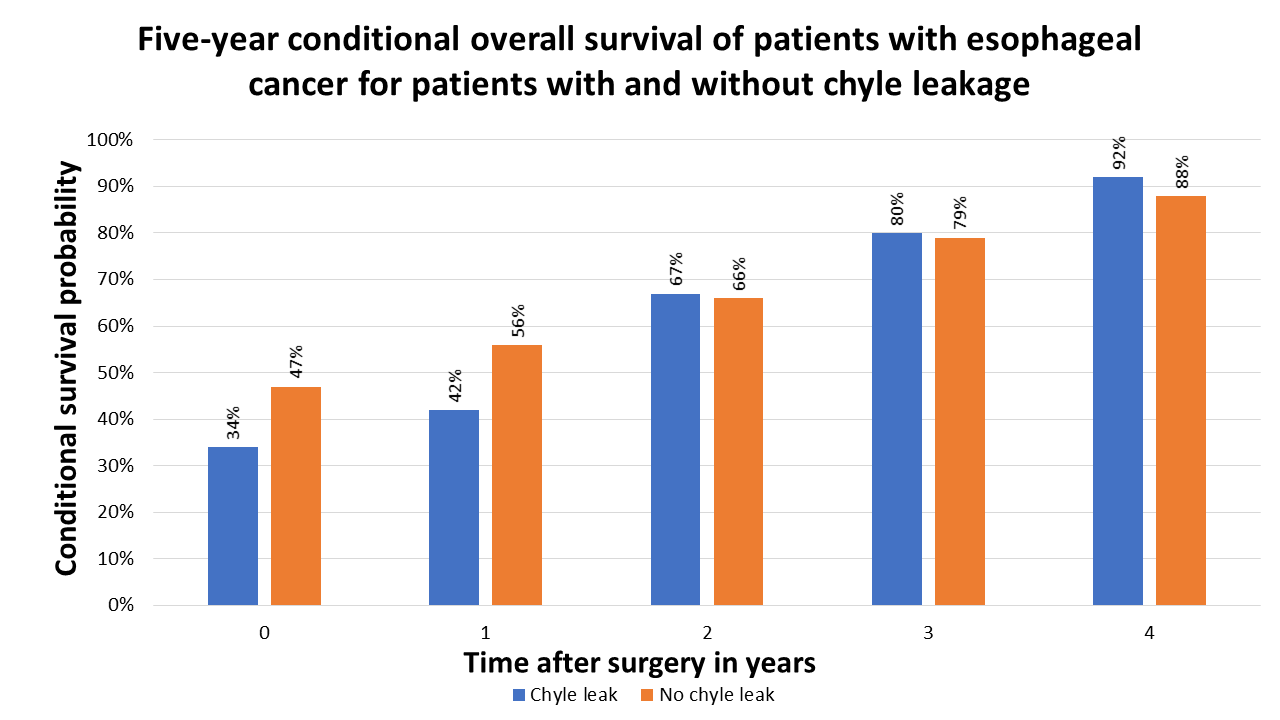
**

**Fig S2. Calibration plot for nonogram. Point estimates for 5-year survival are shown. Error bars are 95 per cent c.i.**


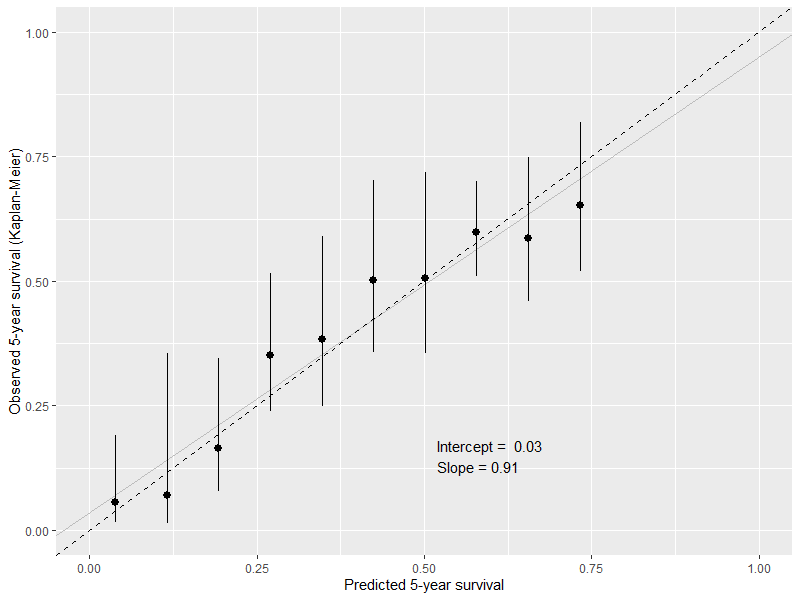

Supplement: Supplementary file 1 — Appendix S1. Supporting Information [file BJS-107-1053-s001.docx]
